# The Association between Nonalcoholic Fatty Pancreas Disease and Diabetes

**DOI:** 10.1371/journal.pone.0062561

**Published:** 2013-05-03

**Authors:** Horng-Yih Ou, Chih-Yuan Wang, Yi-Ching Yang, Ming-Fong Chen, Chih-Jen Chang

**Affiliations:** 1 Department of Internal Medicine, National Cheng-Kung University Hospital, College of Medicine, National Cheng-Kung University, Tainan, Taiwan; 2 Department of Internal Medicine, National Taiwan University Hospital, College of Medicine, National Taiwan University, Taipei, Taiwan; 3 Department of Family Medicine, National Cheng-Kung University Hospital, College of Medicine, National Cheng-Kung University, Tainan, Taiwan; Institute of Medical Research A Lanari-IDIM, University of Buenos Aires-National Council of Scientific and Technological Research (CONICET), Argentina

## Abstract

**Background:**

Fatty infiltration of the pancreas has been shown to interfere with insulin secretion. Both insulin sensitivity and secretion are important in the pathogenesis of diabetes and prediabetes. However, the relationship between diabetes, prediabetes, and fatty pancreas remains unknown. We aim to investigate the relationships that fatty pancreas and nonalcoholic fatty liver disease (NAFLD) have with prediabetes and diabetes in a Chinese population.

**Patients and Methods:**

This was a cross-sectional study. A total of 7,464 subjects were recruited. NAFLD and fatty pancreas were assessed by sonography. Clinico-metabolic parameters were compared among subjects with normoglycemia, prediabetes, and diabetes. Multinomial logistic regression was used to evaluate the relationship between fatty pancreas and NAFLD and diabetes or prediabetes with adjustment for cardiometabolic risk factors.

**Results:**

With an increase in glycemia, a significantly greater proportion of subjects had NAFLD and fatty pancreas (test for trend *p*<0.05). Similar trends were also found for hypertension, general and central obesity, low-HDL cholesterol, and hypertriglyceridemia. In the logistic regression analysis, age, hypertension, male gender, hypertriglyceridemia, and central obesity were significantly associated with prediabetes and diabetes. Furthermore, the ORs of prediabetes and diabetes for NAFLD were 1.798 (95% CI 1.544–2.094) and 2.578 (95% CI 2.024–3.284), respectively. In addition, fatty pancreas was independently related to diabetes (OR, 1.379; 95% CI, 1.047–1.816) and prediabetes (OR, 1.222; 95% CI, 1.002–1.491) in male subjects.

**Conclusions:**

Both NAFLD and fatty pancreas were associated with diabetes independent of age, gender, adiposity, and other cardiometabolic risk factors. Fatty pancreas was also related to prediabetes in males.

## Introdution

There is an increasing prevalence of obesity in many countries around the world, and this has significant adverse health and economic effects [1]. In susceptible human subjects, a positive energy balance and impaired lipid storage capacity of the subcutaneous fat with regard to storing excess energy can result in excess visceral adiposity and increased accumulation of fat in undesired sites (i.e., ectopic fat deposition) such as the liver, skeletal muscle, heart, and even in the pancreas [2]–[3]. Both visceral fat and ectopic fat deposition play important roles in the pathogenesis of obesity-related metabolic consequences, such as diabetes, hypertension, cardiovascular disease, and cancer. Several recent studies suggest that, in both the general population and subjects with coronary artery disease, indices of abdominal obesity are better predictors of all-cause and cardiovascular mortality than body mass index (BMI) [4]–[5]. In addition, nonalcoholic fatty liver disease (NAFLD) is associated with insulin resistance, type 2 diabetes, metabolic syndrome, atherosclerosis [6]–[7], and a greater risk of cardiovascular events [8]. NAFLD can thus be considered an early predictor of metabolic disorders, even in the normal-weight population [9], and early detection of NAFLD could have benefits in clinical practice.

Ectopic fat deposition in the pancreas (fatty pancreas), also termed “nonalcoholic fatty pancreas disease” (NAFPD) [10], has recently gained much attention. Fatty pancreas may promote the development of chronic pancreatitis [11]–[12] and pancreatic cancer [10] and exacerbate the severity of acute pancreatitis [11]–[12]. Moreover, fatty pancreas facilitates the dissemination and lethality of pancreatic cancer [13], and the formation of pancreatic fistula after pancreatic surgery [14]–[15]. Both pancreatic fat replacement with acinar cell death and pancreatic fat infiltration due to obesity contribute to pancreatic steatosis [11]. In addition, fatty pancreas has been suggested to have a role in type 2 diabetes mellitus. In rats, chronic exposure to a high-fat diet induces both interlobular and intralobular fat accumulation, inflammatory cell infiltration, and fibrosis in the pancreas, and thus damage to the normal pancreatic architecture and islets [16]. Likewise, C57BL/6 mice fed a high fat diet (HFD) develop insulin resistance and features of both NAFLD and fatty pancreas [17]–[18]. In human studies, pancreatic fat content is closely associated with BMI [19], insulin resistance [20]–[21], metabolic syndrome [19]–[20], and hepatic fat content [19], [21]–[23]. However, reports on the relationship between fatty pancreas and β-cell function are inconsistent. Some studies indicate that pancreatic lipid content is negatively associated with insulin secretion in nondiabetic subjects [24] or individuals with prediabetes [25], while others suggest that there is no relationship between β-cell function and pancreatic fat in prediabetic [26] or diabetic subjects [24]. However, there have been no large-scale human studies to examine the independent role of fatty pancreas in impaired glucose metabolism. Therefore, the aim of this study was to investigate the association of fatty pancreas, NAFLD, and metabolic risk factors with prediabetes and diabetes in a Chinese population.

## Patients and Methods

This is a retrospective work in which study subjects were recruited from examinees who finished a physical checkup at the Health Management Center of the National Taiwan University Hospital (NTUH) between January 2009 and December 2009. The study protocol was approved by the Institutional Review Board of NTUH.

After an overnight 12-h fast, all subjects received a blood test, including complete blood count, routine biochemistry, and fasting plasma glucose. Waist circumference (WC) was measured and body mass index (BMI, in kg/m^2^) was calculated, and a BMI ≥25 kg/m^2^ was defined as obese. Central obesity was defined as WC ≥90 cm in males and ≥80 cm in females. Habitual physical exercise was categorized as “regular physical exercise” (vigorous exercise at least three times per week) and “no regular physical exercise”. Cigarette smoking was categorized as “current smokers” (at least one pack per month, lasting for half a year) and “nonsmokers”; and alcohol consumption as “drinkers” (at least one drink per week, lasting for half a year) and “nondrinkers”.

Systolic and diastolic blood pressures (SBP and DBP, respectively) were recorded in the supine position. Hypertension was defined as SBP≥140 mm Hg or DBP≥90 mm Hg, or a documented history of hypertension. Blood glucose was measured by a hexokinase method (Roche Diagnostic GmbH, Mannheim, Germany). Diabetes was defined according to the American Diabetes Association’s recommendation [27]. Prediabetes was defined as fasting plasma glucose ≥5.5 mmol/L but <7 mmol/L. Serum total cholesterol, triglycerides, and high density lipoprotein cholesterol (HDL cholesterol) levels were determined in the central laboratory of National Taiwan University Medical Center with an autoanalyzer (Hitachi 747E, Tokyo, Japan). Low density lipoprotein cholesterol (LDL cholesterol) was calculated using the Friedewald formula. Low-HDL cholesterol was defined as HDL-cholesterol <1.0 mmol/L for males or <1.3 mmol/L for females, and hypertriglyceridemia was defined as triglyceride ≥1.7 mmol/L.

Liver and pancreas sonography were performed simultaneously by a single experienced radiologist with high resolution ultrasonography (HPM2410A, Hewlett Packard, Andover, MA, USA) using a 3.5 MHz linear transducer. Both NAFLD and fatty pancreas were diagnosed by hepatologists who were blind to all the subjects’ medical information. The NAFLD diagnostic criteria included characteristic echo patterns of hepatorenal echo contrast, bright liver, deep (posterior beam) attenuation, and vascular blurring. Fatty pancreas was diagnosed when there was an increase in echogenicity of the pancreatic body over that of the kidney. As the pancreas could not be compared directly with the kidney in the same window, the examiner compared the difference between hepatic and renal echogenicity, and between hepatic and pancreatic echogenicity, to obtain an objective pancreato-renal echo contrast. Using this method, all subjects were classified into either fatty pancreas or non-fatty pancreas groups. The mean inter-observer percentage of agreement for ultrasound diagnosis of fatty pancreas was 72% (κ = 0.63).

Subjects with the following conditions or diseases were excluded: 1) an age of <18 or ≥80 years; 2) a BMI of ≥35 kg/m^2^; 3) alcohol consumption ≥20 g/day in the last year; 4) serum creatinine >133 µmol/L; 5) anemia and known hemoglobinopathy; 6) history of recent surgery, trauma, illness, bleeding, or transfusion within the last 6 months; 7) a past medical history of diabetes, or having previously been treated with insulin or an antidiabetic agent; 8) any acute or chronic inflammatory disease, as determined by a leukocyte count >10,000/mm^3^ or clinical signs of infection; and 9) any other major diseases, including generalized inflammation or advanced malignant diseases contraindicating this study.

### Statistical Analyses

SPSS software (version 17.0; SPSS, Chicago, IL) was used for the statistical analyses. All normally distributed continuous variables were expressed as means ± SD. Study subjects were divided into three groups based on the glycemic status: normal, prediabetes, and diabetes. The continuous variables among the groups were compared using ANOVA, or a Kruskal–Wallis test if the distribution was not normal. χ2 tests were used to analyze differences in categorical variables among groups. Multinomial logistic regression was used to evaluate the relationships between fatty parncreas and NAFLD and diabetes or prediabetes, with adjustment for cardiometabolic risk factors. A *p-* value less than 0.05 was considered statistically significant.

## Results

**Figure 1:**
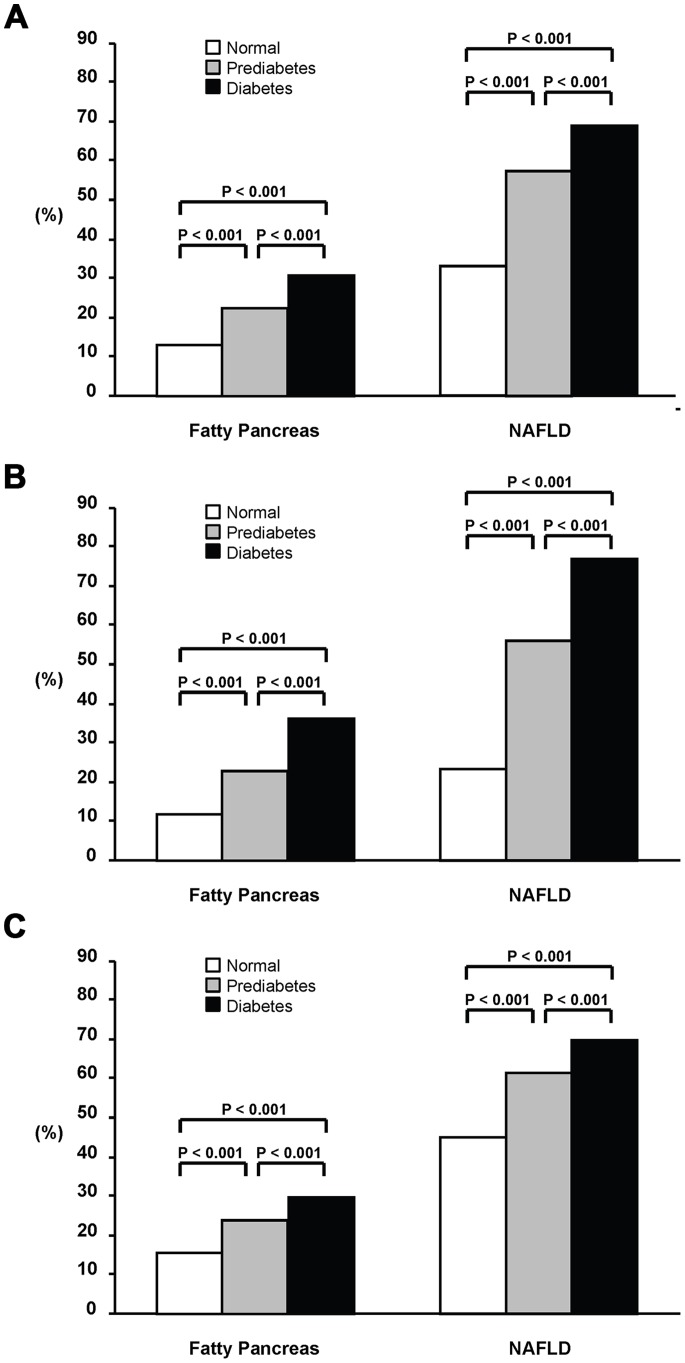
The proportions of fatty pancreas and NAFLD in all the subjects (A), females (B), and males (C) with normoglycemia, prediabetes, and diabetes.

In the final analysis, a total of 7,464 subjects were included and classified into normal (*n* = 5,756, 77%), prediabetes (*n* = 1,225, 16%), and diabetes (*n* = 483, 6%) groups. Table 1 shows a comparison of the clinical characteristics among the groups based on gender-stratified analysis. There were significant differences in age,WC, BMI, systolic/diastolic blood pressure, fasting plasma glucose, A1C, alanine transaminase (ALT), aspartate transaminase (AST), ALT/AST ratio, total cholesterol, triglyceride, HDL-cholesterol, and LDL cholesterol for both genders. Male subjects had a significantly higher prevalence of NAFLD (50.2% vs 29.9%, p<0.001) and fatty pancreas (18.1% vs 14.2%, p<0.001) than female ones. As seen in Figure 1, with an increase in glycemia, a significantly greater proportion of subjects of both genders had NAFLD and fatty pancreas (*p*<0.001, test for trend), and similar trends were also found for hypertension, general obesity, central obesity, low-HDL cholesterol, and hypertriglyceridemia. However, there were no significant differences in the lifestyle factors among the groups, such as alcohol consumption and smoking.

**Table 1:** Clinical characteristics of study subjects with normoglycemia, prediabetes, and diabetes stratified by gender.

		Female				Male		
	Normoglycemia	Prediabetes	Diabetes	*P*	Normoglycemia	Prediabetes	Diabetes	*P*
*n*	2326	390	155		3120	835	328	
Age (years)	50±11	57±10	61±8	<0.001	50±11	56±10	59±9	<0.001
Waist circumference (cm)	81.3±8.1	86.9±8.5	90.3±8.8	<0.001	86.7±7.7	89.4±7.7	90.7±8.0	<0.001
BMI (kg/m^2^)	22.4±2.9	24.6±3.4	25.4±3.3	<0.001	24.5±2.8	25.4±2.9	25.6±3.0	<0.001
Systolic blood pressure (mmHg)	112±14	124±15	129±14	<0.001	120±14	127±15	126±14	<0.001
Diastolic blood pressure (mmHg)	66±9	73±9	74±8	<0.001	71±9	75±10	74±9	<0.001
Fasting plasma glucose (mmol/L)	4.9±0.3	5.9±0.3	7.9±2.0	<0.001	5.0±0.3	5.9±0.3	7.9±1.9	<0.001
A1C (%)	5.4±0.3	5.8±0.3	7.4±1.3	<0.001	5.4±0.3	5.8±0.3	7.3±1.2	<0.001
AST (U/L)	22±8	24±9	28±16	<0.001	26±11	27±11	29±18	<0.001
ALT (U/L)	21±13	27±16	34±24	<0.001	32±22	34±22	40±43	<0.001
ALT/AST ratio	0.9±0.3	1.1±0.3	1.2±0.3	<0.001	1.2±0.4	1.3±0.4	1.3±0.4	<0.001
Creatinine (µmol/L)	74±9	74±10	75±11	0.027	96±10	96±11	96±12	0.193
Total cholesterol (mmol/L)	5.3±0.9	5.7±0.9	5.5±1.0	<0.001	5.3±0.8	5.4±0.9	5.1±1.0	<0.001
Triglyceride (mmol/L)*	1.0±0.6	1.3±0.7	1.7±0.9	<0.001	1.5±1.0	1.7±1.0	1.8±1.2	<0.001
HDL cholesterol (mmol/L)	1.5±0.3	1.4±0.3	1.3±0.3	<0.001	1.2±0.3	1.2±0.2	1.1±0.3	<0.001
LDL cholesterol (mg/dL)	3.0±0.8	3.3±0.8	3.0±0.9	<0.001	3.1±0.8	3.3±0.8	3.0±0.9	<0.001
Hypertension (%)	3.4	13.6	21.3	<0.001	7.7	17.1	14.3	<0.001
General obesity (%)	17.3	42.8	54.2	<0.001	40.4	53.3	56.1	<0.001
Central obesity (%)	55.1	80.2	89.7	<0.001	32.8	44.9	54.3	<0.001
Low-HDL cholesterol (%)	30.7	45.4	59.4	<0.001	30.8	32.7	47.0	<0.001
Hypertriglyceridemia (%)	10.2	23.8	41.3	<0.001	27.6	37.6	42.4	<0.001
Regular physical exercise(≥3 times/wk)	24.8	28.5	31.6	0.062	29.9	39.3	39.6	<0.001
Current smoking (%)	2.8	1.5	2.6	0.327	18.2	15.9	18.0	0.318
Current alcohol drinking (%)	3.3	2.6	3.2	0.743	15.0	19.2	14.3	0.05

Data expressed as means ± SD. ALT: alanine transaminase; AST: aspartate transaminase; BMI: body mass index; A1C: glycosylated hemoglobin; HDL: high-density lipoprotein; LDL: low-density lipoprotein; NS: not significant.

* Kruskal–Wallis test.

Table 2 shows the effects of clinical variables on the risk of prediabetes and diabetes based on the results of the multinomial logistic regression. The results show that age, ALT/AST ratio, hypertension, male gender, hypertriglyceridemia, and central obesity were significantly associated with prediabetes and diabetes. Furthermore, the ORs of prediabetes and diabetes for NAFLD were 1.798 (95% CI 1.544–2.094) and 2.578 (95% CI 2.024–3.284), respectively. However, fatty pancreas was only associated with an increased risk for diabetes (OR, 1.344; 95% CI, 1.074–1.682), and not prediabetes (OR, 1.134; 95% CI, 0.962–1.337). The results remained the same when substituting central obesity with general obesity in the multinomial regression analysis (data not shown). As there were significant gender differences in the prevalence of NAFLD and fatty pancreas, we further performed a gender-stratified analysis. Among males subjects, both NAFLD and fatty pancreas were associated with increased risk of diabetes (OR, 2.128; 95% CI, 1.589–2.850 and OR, 1.379; 95% CI, 1.047–1.816, respectively) and prediabetes (OR, 1.609; 95% CI, 1.335–1.939 and OR, 1.222; 95% CI, 1.002–1.491, respectively)(Table 3). In contrast, only NAFLD was associated with diabetes (OR, 3.771; 95% CI, 2.414–5.889) and prediabetes (OR, 2.150; 95% CI, 1.652–2.798) in female subjects (Table 4).

**Table 2:** Logistic regression analysis for factors associated with prediabetes and diabetes in study subjects.

	Prediabetes			Diabetes		
	OR	95% CI	*P* value	OR	95% CI	*P* value
Age (years)	1.058	1.050 ∼ 1.065	<0.001	1.105	1.092 ∼ 1.117	<0.001
Creatinine	1.001	0.995 ∼ 1.007	0.774	0.999	0.990 ∼ 1.008	0.846
ALT/AST ratio	1.801	1.477 ∼ 2.197	<0.001	2.726	2.042 ∼ 3.639	<0.001
Hypertension, yes vs. no	1.895	1.552 ∼ 2.314	<0.001	1.593	1.199 ∼ 2.116	0.001
Gender, female vs. male	0.738	0.596 ∼ 0.913	0.005	0.724	0.527 ∼ 0.995	0.046
NAFLD, yes vs. no	1.798	1.544 ∼ 2.094	<0.001	2.578	2.024 ∼ 3.284	<0.001
Fatty pancreas, yes vs. no	1.134	0.962 ∼ 1.337	0.134	1.344	1.074 ∼ 1.682	0.010
Low HDL-cholesterol, yes vs. no	0.962	0.831 ∼ 1.113	0.600	1.443	1.167 ∼ 1.786	0.001
Hypertriglyceridemia, yes vs. no	1.379	1.179 ∼ 1.612	<0.001	1.586	1.269 ∼ 1.981	<0.001
Central obesity, yes vs. no	1.208	1.042 ∼ 1.402	0.012	1.350	1.072 ∼ 1.699	0.011
Current smoking, yes vs. no	0.994	0.802 ∼ 1.233	0.958	0.743	0.544 ∼ 1.016	0.063
Current alcohol drinking, yes vs. no	0.613	0.374 ∼ 1.003	0.051	0.735	0.329 ∼1.642	0.453
Regular physical exercise, yes vs. no	0.900	0.778 ∼ 1.040	0.153	0.893	0.719 ∼ 1.108	0.302

NAFLD: nonalcoholic fatty liver disease.

**Table 3:** Logistic regression analysis for factors associated with prediabetes and diabetes in female subjects.

	Prediabetes			Diabetes		
	OR	95% CI	*P* value	OR	95% CI	*P* value
Age (years)	1.061	1.048 ∼ 1.075	<0.001	1.089	1.066 ∼ 1.112	<0.001
Creatinine	0.997	0.985 ∼ 1.010	0.681	1.007	0.989 ∼ 1.025	0.474
ALT/AST ratio	2.855	1.956 ∼ 4.169	<0.001	4.673	2.714 ∼8.046	<0.001
Hypertension, yes vs. no	2.287	1.557 ∼ 3.361	<0.001	3.070	1.875 ∼ 5.028	<0.001
NAFLD, yes vs. no	2.150	1.652 ∼ 2.798	<0.001	3.771	2.414 ∼ 5.889	<0.001
Fatty pancreas, yes vs. no	0.932	0.693 ∼ 1.253	0.641	1.293	0.870 ∼ 1.922	0.203
Low HDL-cholesterol, yes vs. no	1.161	0.904 ∼ 1.492	0.243	1.372	0.930 ∼2.022	0.111
Hypertriglyceridemia, yes vs. no	1.298	0.956 ∼ 1.761	0.094	1.988	1.326 ∼ 2.980	0.001
Central obesity, yes vs. no	1.385	1.030 ∼ 1.863	0.031	1.534	0.860 ∼ 2.738	0.147
Current smoking, yes vs. no	1.377	0.563 ∼ 3.367	0.483	0.770	0.245 ∼2.422	0.655
Current alcohol drinking, yes vs. no	0.449	0.093 ∼ 2.165	0.318	0.144	0.022 ∼0.949	0.044
Regular physical exercise, yes vs. no	1.156	0.890 ∼ 1.501	0.278	1.019	0.687 ∼ 1.511	0.927

NAFLD: nonalcoholic fatty liver disease.

**Table 4:** Logistic regression analysis for factors associated with prediabetes and diabetes in male subjects.

	Prediabetes			Diabetes		
	OR	95% CI	*P* value	OR	95% CI	*P* value
Age (years)	1.053	1.044 ∼ 1.062	<0.001	1.105	1.090 ∼ 1.121	<0.001
Creatinine	1.003	0.996 ∼ 1.010	0.431	0.998	0.987 ∼ 1.009	06946
ALT/AST ratio	1.542	1.219 ∼ 1.952	<0.001	2.309	1.637 ∼ 3.257	<0.001
Hypertension, yes vs. no	1.754	1.388 ∼ 2.215	<0.001	1.189	0.833 ∼ 1.695	0.340
NAFLD, yes vs. no	1.609	1.335 ∼ 1.939	<0.001	2.128	1.589 ∼ 2.850	<0.001
Fatty pancreas, yes vs. no	1.222	1.002 ∼ 1.491	0.048	1.379	1.047 ∼ 1.816	0.022
Low HDL-cholesterol, yes vs. no	0.857	0.714 ∼ 1.028	0.096	1.434	1.109 ∼ 1.855	0.006
Hypertriglyceridemia, yes vs. no	1.431	1.193 ∼ 1.718	<0.001	1.458	1.116 ∼ 1.904	0.006
Central obesity, yes vs. no	1.116	0.978 ∼ 1.389	0.087	1.369	1.058 ∼ 1.771	0.017
Current smoking, yes vs. no	0.967	0.774 ∼ 1.208	0.767	0.722	0.522 ∼ 1.001	0.051
Current alcohol drinking, yes vs. no	0.664	0.396 ∼ 1.113	0.120	0.976	0.395 ∼2.414	0.959
Regular physical exercise, yes vs. no	0.810	0.679 ∼ 0.966	0.019	0.845	0.650 ∼ 1.097	0.206

NAFLD: nonalcoholic fatty liver disease.

## Discussion

To the best of our knowledge, this is the first study to investigate the independent role of fatty pancreas in prediabetes and diabetes in a large cohort. Our results suggest that both NAFLD and fatty pancreas were strongly associated with diabetes and prediabetes after adjustment for age, adiposity, ALT/AST ratio, and other cardiometabolic risk factors in male subjects. However, in female subjects, only NAFLD was associated with diabetes and prediabetes.

An association between ectopic fat accumulation in the liver and pancreas has been previously observed in many [19]–[23], but not all [25], [28], studies (Table 5). Recently, Rossi *et al.* showed that visceral adipose tissue (VAT), measured with MRI, is the main predictor of ectopic fat deposition in both liver and pancreas. In obese subjects, women had significantly lower pancreas fat than men. Taken together, 59.2% and 46% of the variance of pancreas and liver fat content was explained by gender and VAT [29], respectively. Using MR imaging to evaluate pancreatic fat content, two small studies by Schwenzef *et al.* [28] (*n* = 16) and Heni *et al.* [25] (*n* = 51) that examined nondiabetic subjects reported no correlation between pancreatic and hepatic fat content. In contrast, Al-Haddad *et al.* [23] and Sepe *et al.* [19] found that in subjects undergoing EUS both fatty liver and increased BMI were independent predictors of fatty pancreas. Notably, when compared with Schwenzef *et al.* and Heni *et al.*, these two studies were larger (*n* = 120 and 230, respectively), the subjects were older (mean 63–65 years), and diabetic patients were not excluded. In addition, the positive correlation of NAFLD and fatty pancreas was also demonstrated in another three studies using transabdominal ultrasound [20], histopathological findings from postmortem collected material [22], and proton magnetic resonance spectroscopy (^1^H MRS) [21].

**Table 5:** Summary of studies investigating the relationships among ectopic fat accumulation in liver, pancreas, and visceral adipose tissue.

First author [Ref.]	Study design and population	NAFLD diagnosis	FP diagnosis	No. of subjects	Age	BMI	Results
Schwenzer *et al.*,(2008) [28]	Cross-sectional design; Nondiabetic obese/overweight subjects	Two established MR imaging techniques	Two established MR imaging techniques	16	50.4	31.7	No correlation between pancreatic and hepatic fat content by either MR imaging techniques
Al-Haddad *et al*.,(2009) [23]	Retrospective design;Case-control; Patientsundergoing EUS	CT or MRI or transabdominal US or EUS	EUS	60/60 (FP/non-FP)	65/66	31.7/25.4	Hepatic steatosis, alcohol (>14 g/wk), and increased BMI are predictors of hyperechogenic pancreas
Lee *et al.*, (2009) [20]	Retrospective design;Case-control; Nondiabeticsubjects		Transabdominal US	180/113 (FP/non-FP)	45.4/44.4	26.5/24.4	HOMA-IR, visceral fat (by CT scan), and ALT independently related to fatty pancreas
Heni *et al.*,(2010) [25]	Cross-sectional design; Nondiabetic subjects	MRS	MRI	28/23 (NGT vs. IFG and/or IGT)	43.1/52.9	29.6/30.3	Pancreatic fat content is associated with 1. BMI, VAT, and waist circumference, but not with hepatic fat content. 2. Impaired insulin secretion in IFG/IGT, but not NGT
van Geenen *et al.*,(2010) [22]	Autopsy	Pathology (NAFLD activity score)	Pathology (pancreatic steatosis score)	80	68	26	1. Presence of intralobular pancreatic fat is related to nonalcoholic steatohepatitis. 2. Total pancreatic fat is related to NAFLD.
Hannukainen *et al.*, (2011) [21]	Cross-sectional design; Healthy monozygotic male twin pairs	1H MRS	1H MRS	8/8 (More active/less active)	25.8	24.3/25.1	1. Hepatic fat, but not pancreatic fat, is lower in more physically active subjects. 2. Pancreatic fat content is positively associated with hepatic fat content and insulin resistance
Sepe *et al.*, (2011) [19]	Prospective design;Case-control; Patientsundergoing EUS;	EUS	EUS	64/166 (FP/non-FP)	62.6/62.1	29.4/26.5	1. Fatty pancreas is independently associated with fatty liver (OR 3.61) and BMI (OR 1.05). 2. No association between increasing age and prevalence of fatty pancreas
Rossi *et al.*, (2011) [29]	Cross-sectional design; Nondiabetic subjects	MRI	MRI	12/18/20 (Lean/obese men/obese women)	47.3/45.8/52.1	22.9/35.4/34.6	1. 59.2% and 46% of the variance in pancreas and liver fat content is explained by gender and VAT, respectively. 2. Insulin resistance is associated with liver but not with pancreas lipid content.

EUS: endoscopic ultrasound; FP: fatty pancreas;

1 H MRS: proton magnetic resonance spectroscopy; MRI: magnetic resonance imaging; MRS: Magnetic resonance spectroscopy; US: ultrasound; VAT: visceral adipose tissue.

In spite of their significant association, whether NAFLD and fatty pancreas have independent effects on the glucose metabolism remains unknown. In addition, it has been convincingly demonstrated that both obesity and NAFLD are closely associated with impaired glucose metabolism, and we found that after adjustment for obesity and NAFLD, fatty pancreas still remained positively associated with diabetes (OR, 1.379; 95% CI, 1.047–1.816) and prediabetes (OR, 1.222; 95% CI, 1.002–1.491) in male subjects. However, we did not observe a similar relationship in female subjects. The lack of this association in women could be explained by the gender difference in ectopic fat deposition in liver and pancreas, as shown in the current study and others [29]–[30]. Another explanation could be attributable to type 2 error, due to the lower prevalence of fatty pancreas and lower sensitivity in the ultrasonographic diagnosis of fatty pancreas compared to fatty liver. Of particular note is the fact that, in contrast to NAFLD, the pathophysiological mechanisms and clinical relevance of fatty pancreas are less clear. Previous studies have indicated that fatty infiltration of the pancreas contributes to a loss of β-cell mass and function [31]–[32], which possibly leads to the development of diabetes [33]. In obese Zucker diabetic fatty rats, the triacylglycerol content of islets in prediabetic rats increased significantly and preceded the development of diabetes [34]. However, in human studies the data are inconsistent. Tushuizen *et al.* found that pancreatic fat, as measured by magnetic resonance spectroscopy (MRS), was negatively associated with oral glucose tolerance test-based measures of insulin secretion in nondiabetic subjects [24]. In addition, the results of the regression analysis in Heni *et al.* indicated that pancreatic fat was a stronger determinant of impaired insulin secretion than visceral fat in subjects with prediabetes, but not in those with normoglycemia [25]. In contrast, Le *et al.* found that there were no significant correlations between pancreatic fat fraction and the markers of β-cell function in obese adolescents and young adults [35]. Another work that used the gold standard hyperglycemic clamp also found no relation between pancreatic fat content and β-cell function in subjects with impaired glucose metabolism [26]. In addition, Tushuizen *et al.* reported no association between pancreatic fat and β-cell dysfunction in diabetic patients, and suggested that once diabetes occurs, factors additional to pancreatic fat account for further declines in β-cell functioning [24]. It is likely that these discrepancies with regard to the relationship between pancreatic fat and β-cell dysfunction found in these works may be due to differences in the methods by which fatty infiltration of the pancreas was estimated, and the glycemic status of the study subjects. In our study, using a relatively large number of subjects, we demonstrated that fatty pancreas was independently associated with diabetes and prediabetes in males. This result implies that in addition to insulin resistance associated with obesity and ectopic fat deposition, such as NAFLD, the impaired β-cell function in fatty pancreas may contribute to the further development of diabetes, as β-cell dysfunction is essential for the development of type 2 diabetes [36]. In addition, once diabetes develops, fatty replacement of damaged tissue may contribute to the extra-islet pancreatic fat deposition [24]. Moreover, the increased levels of malonyl-CoA caused by hyperglycemia in diabetes also inhibit carnitine palmitoyltransferease-1, leading to a decrease in mitochondrial β-oxidation and further stimulation of intracellular triglyceride accumulation [37].

In the current study, subjects with NAFLD had a significantly higher risk of prediabetes (OR 1.798, 95% CI 1.544–2.094) or diabetes (OR 2.578, 95% CI 2.024–3.284) compared to those without it. This finding is compatible with the notion that NAFLD is an early precursor of diabetes/prediabetes and full-brown metabolic syndrome [38]–[40], and many previous studies have shown that NAFLD was independently associated with diabetes [41]–[44]. Surrogate markers of NAFLD, including γ-glutamyltransferase (GGT) [42] and ALT [44] or fatty liver indices [43], have been shown to be predictive of diabetes [42]–[44] in prospective studies. Furthermore, a meta-analysis study also showed that ultrasonography-diagnosed NAFLD was associated with more than a doubling (pooled relative risk 2.52) in the risk of incident diabetes [44]. Consequently, the occurrence of diabetes might induce progressive liver damage, including cirrhosis [45], increased risk of developing hepatocellular carcinoma [46], and death [47]. In addition to diabetes, two studies showed that liver enzyme activities and fatty liver indices are also associated with the incidence of prediabetes. Nannipieri *et al.* reported that raised GGT is an independent predictor of impaired glucose tolerance at seven years follow-up in subjects with normal glucose tolerance [42]. In a prospective study of a healthy Japanese cohort, Suzuki *et al.* reported the development of nonalcoholic hypertransaminasemia (as a surrogate of NAFLD) followed weight gain and low HDL cholesterolemia in chronological order, and preceded glucose intolerance [40]. Recently, Ruckert *et al.*, in a population-based health survey, found that elevated GGT values, as well as fatty liver index, are significantly associated with both prediabetes and diabetes [48]. Consistent with that study, we further confirmed that ultrasonography-diagnosed NAFLD is an independently associated factor of both prediabetes and diabetes after adjustment for ALT/AST ratio and other confounding factors. This link between NAFLD and impaired glucose metabolism may be related to insulin resistance. In humans, a strong relationship exists between fat accumulation in the liver and whole-body insulin resistance [49]. In addition, one previous study among Pima Indians found that GGT was associated with both hepatic insulin resistance and later declines in hepatic insulin sensitivity in nondiabetic subjects, as well as the development of type 2 diabetes [41]. On the other hand, insulin resistance may enhance hepatic fat accumulation by increasing free fatty acid delivery, and then stimulate the anabolic process due to hyperinsulinemia [50]. Therefore, it is conceivable that prediabetes/diabetes and insulin resistance could develop at the same time as NAFLD.

Older age and male gender were significantly associated with diabetes and prediabetes in the present study. These findings are compatible with a previous national survey of Taiwanese residents in 2002, which showed a male preponderance and significant age-trend in the prevalence of diabetes [51]. Furthermore, the associations of hypertension, low-HDL cholesterol, and hypertriglyceridemia with diabetes are in line with previous research showing that metabolic syndrome and its components are significantly associated with diabetes [52].

There are some limitations to this work, as follows. First, since this study used a cross-sectional design, it does not allow causal inferences between fatty pancreas, NAFLD and diabetes or prediabetes, but the causal relationship is expected to link NAFLD and fatty pancreas with prediabetes/diabetes, not vice versa. Second, the diagnoses of fatty pancreas and NAFLD were made by sonography, but not confirmed pathologically. Although magnetic resonance-based techniques are frequently used for measurement of pancreatic fat content**,** they are difficult to perform in clinical practice. On the other hand, abdominal sonography is an established non-invasive tool used as a screening modality, which has been shown to be accurate and cost-effective in diagnosing fatty pancreas in previous cohort studies [20], [53]. More importantly, increased deposition of fat, which has infiltrated along the pancreatic septa, has been shown to be a major determining factor of pancreatic echogenicity [54]. The results of these earlier works support the use of sonography in the present study. However, the major drawbacks of sonography include operator dependency and insensitivity with regard to small amounts of fat. To minimize the inter-observer variability, the sonography in this work was performed by a single experienced radiologist. Furthermore, the ultrasonography data were interpreted by hepatologists who were blind to the subjects’ past history or biochemical results to further reduce potential bias. Third, although insulin sensitivity and secretion are important in the pathogenesis of diabetes and prediabetes, we regret that we did not measure the subjects’ insulin levels, because this is not part of the routine physical checkup in our institution, and we did not have the serum for this measurement. Instead, we adjusted components of metabolic syndrome as a proxy for insulin resistance. Finally, because the racial differences in the fatty pancreas have not been reported yet, more studies on this topic are needed.

In conclusion, in this work we found that both fatty pancreas and NAFLD are important associated factors of newly diagnosed diabetes independent of age, gender, adiposity, and other cardiometabolic risk factors in a large Chinese cohort. In addition, fatty pancreas was independently related to prediabetes in male subjects, but not female ones.
